# An Evidence-Based Framework for Patient-Facing Artificial Intelligence Integration in the Emergency Department

**DOI:** 10.1016/j.acepjo.2026.100466

**Published:** 2026-07-09

**Authors:** Tehreem Rehman, Philip Jarrett, Joshua Lesko, James Augustine, Bradley D. Shy, Rohit B. Sangal, Nicholas Genes, Donald U. Apakama, Ethan E. Abbott, Abhi Mehrotra, Richard Andrew Taylor

**Affiliations:** 1 Department of Emergency Medicine, Icahn School of Medicine at Mount Sinai, New York, New York, USA; 2 Department of Emergency Medicine, University of Texas Southwestern Medical Center, Dallas, Texas, USA; 3 Eastern Virginia Medical School at ODU Emergency Medicine, Norfolk, Virginia, USA; 4 Department of Emergency Medicine, Wright State University, Dayton, Ohio, USA; 5 Department of Emergency Medicine, University of Colorado School of Medicine, Aurora, Colorado, USA; 6 Department of Emergency Medicine, Yale University, New Haven, Connecticut, USA; 7 Ronald O. Perelman Department of Emergency Medicine, NYU Grossman School of Medicine, New York, New York, USA; 8 Artificial Intelligence and Human Health, Icahn School of Medicine at Mount Sinai, New York, New York, USA; 9 Department of Emergency Medicine, University of North Carolina, Chapel Hill, North Carolina, USA; 10 Department of Emergency Medicine, University of Virginia, Charlottesville, Virginia, USA

**Keywords:** patient-facing artificial intelligence, emergency medicine, large language models, human oversight, health equity, natural language processing, patient safety

## Abstract

The emergency department is a high-need setting for patient-facing artificial intelligence (PF-AI) because it functions as both a health care safety net and, increasingly, a digital front door for unscheduled acute care. We define PF-AI as tools that interact directly with patients to collect information, deliver tailored communication, and support decision making under clinician governance. This article presents a constrained framework for where PF-AI may add value across the emergency care journey—including structured intake, patient education, multilingual communication, and postdischarge follow-up—and where strict boundaries are required given risks of undertriage, confabulated content, workflow burden, privacy harm, inequitable access, cost escalation, and medico-legal ambiguity. We map operational touchpoints from prearrival to follow-up, specify information-flow and accountability checkpoints, and propose a human oversight model with defined escalation roles. The framework emphasizes equity, interoperability, usability, privacy, safety verification, and auditability, recognizing that PF-AI should augment rather than replace clinician judgment or existing non-AI workflows.

## 1 Background

For millions of patients each year, the emergency department (ED) serves as the first point of entry into the health care system.^1^ Many arrive with a fragmented medical record, inconsistent ambulatory care, or limited engagement with digital health tools.^2^, ^3^, ^4^ As a result, the ED functions both as a communication bottleneck and a primary entry point for clinical data. Key history, medications, and contact information are captured and routed into longitudinal records, under pressure and with incomplete context. At the same time, the ED visit contains multiple periods of unavoidable waiting: before triage, while tests are pending, and prior to discharge. These pauses, often experienced as idle or anxiety-provoking, can instead be reframed as opportunities for information capture, education, and patient engagement.

In this manuscript, patient-facing artificial intelligence (PF-AI) is an operational term for patient-interactive digital systems that perform three functions: (1) data transformation, converting patient-reported information into structured records through natural language processing; (2) workflow automation, including asynchronous triage-support and follow-up coordination; and (3) multimodal communication across text, voice, and image modalities. Current evidence for these capabilities is heterogeneous and context-dependent.^5^, ^6^, ^7^, ^8^, ^9^, ^10^

We present PF-AI as a constrained implementation framework, not a universal solution. In many circumstances, simpler alternatives (for example, structured forms, nurse-led protocols, or nonartificial-intelligence digital tools) may be safer or more practical. The key question is not whether artificial intelligence is available, but whether it adds measurable value over existing workflows for specific ED populations and tasks. Recent commercialization of patient-facing health AI platforms and growing consumer wearable signal streams further underscore the need for ED-specific governance and evidence standards.^11^, ^12^, ^13^, ^14^, ^15^, ^16^

Risks are substantial and cluster in three domains. Technical risks include unreliable outputs^17^, ^18^, ^19^ and model bias.^20^ Implementation risks include fragmented integration into ED workflows^21^ and clinician cognitive overload from parallel data streams.^22^ Structural risks include digital- and health-literacy barriers,^23^ the environmental cost of compute-intensive training,^24^ and unresolved liability when recommendations contribute to harm.^25^^,^^26^

This framework is written for emergency clinicians, health-system executives, and clinical informatics teams designing PF-AI programs in emergency care. The evaluative domains used in this framework (equity, interoperability, usability, privacy, safety, and accountability) are grounded in the World Health Organization's ethics and governance guidance for AI in health,^27^ clinical reporting and audit standards, and health-system governance recommendations. We organize applications across six phases of the ED visit (previsit, triage, preclinician evaluation, postevaluation communication, discharge, follow-up) with explicit human oversight expectations at each step.^27^, ^28^, ^29^, ^30^

PF-AI tools must account for the realities of emergency care: time pressure, emotional intensity, and variable digital literacy. Patients in pain, acute anxiety, or cognitive distress process information differently than ambulatory populations, and effective design requires accommodating these states directly.^31^ PF-AI should always be paired with an equivalent staff-assisted fallback, such as interpreter-supported intake, bedside staff engagement, or standard nondigital workflows, so patients who cannot or do not wish to engage digitally are not excluded.^32^^,^^33^

PF-AI is most useful when translating complex medical information into accessible formats. Systems should avoid vague verbal probability terms and instead use consistent numerical denominators and absolute risk differences. Visual risk displays should use part-to-whole formats such as icon arrays, which outperform bar charts and numeric probability alone for conveying risk magnitude to patients with variable numeracy.^34^ Information delivery should also be multimodal: audiovisual media improve comprehension for patients who struggle with dense text, particularly when explaining treatment options or discharge instructions.^35^^,^^36^

Design must account for the diverse cognitive profiles of the ED population. For aging patients or those with limited health literacy, interfaces should prioritize universal symbols and simplified navigation structures that require minimal steps.^23^^,^^37^^,^^38^ Content should be tailored not just to the patient's medical history but to their status in the ED journey, providing timely updates that reduce anxiety without causing information overload.^32^^,^^33^ For patients with cognitive impairment or those overwhelmed by the ED environment, AI tools can act as "scaffolding," breaking down tasks into manageable components and offering step-by-step guidance.^23^

Patients must understand they are interacting with an automated system, not a clinician. PF-AI interfaces should use clear disclosure and nonanthropomorphic design cues to distinguish automated support from clinician judgment. Trust also depends on transparent privacy practices, data provenance, and performance monitoring across demographic groups.^39^ Ransomware attacks that have disrupted emergency care at adjacent hospitals have made health care cybersecurity risks visible to patients themselves.^40^^,^^41^ Because PF-AI tools handle sensitive patient-reported data, explicit safeguards are a precondition for trust, not a downstream assurance.

By codesign, we refer to sustained, iterative partnership with patients and caregivers across problem definition, workflow design, prototype testing, and governance decisions, rather than one-time feedback sessions. Participatory design should intentionally include groups at highest risk of exclusion, so that PF-AI systems do not widen disparities while attempting to improve access.^32^^,^^36^^,^^42^

A central concern is that PF-AI could perpetuate or amplify existing health disparities. When training data reflect historical biases, algorithms perform less accurately for underrepresented groups, including racial minorities and patients with lower socioeconomic status.^20^^,^^43^ Digital technology adoption itself varies by age, race, and ethnicity.^44^ These gaps are most acute for non-English-speaking populations, who face compounded barriers of limited broadband access, lower digital literacy, and a lack of linguistically or culturally tailored interfaces.^9^^,^^44^

Mitigating these risks requires ongoing equity audits, transparent reporting of model performance across demographic subgroups, and active participation of patients and community representatives in both design and evaluation.^28^^,^^45^ Codesign surfaces biases early and keeps PF-AI aligned with patient-centered needs rather than structural assumptions.^32^^,^^46^ Combined with robust privacy protections, explainability, and continuous monitoring for bias and error,^35^^,^^47^ these practices allow PF-AI to strengthen communication and safety without displacing the human connection that defines emergency medicine.

## 2 The Patient-Care Journey: Integrating Patient-Facing AI

Patient-facing AI most clearly adds value when it reduces friction in predictable, guideline-driven touchpoints while preserving clinician authority at decision points. We organize applications across six phases of emergency care—previsit, triage, preclinician evaluation, postevaluation communication, discharge, and follow-up—and apply a uniform test at each: does PF-AI add measurable value over existing workflows, and what human oversight structure is required to use it safely? Table 1 summarizes evidence-based applications across these phases.

**Table 1:** Patient-facing AI applications across the emergency care journey.

Phase	Representative PF-AI applications	Required human oversight
Previsit	Patient-portal and mobile symptom intake; multilingual pre-triage narrative capture; on-route ambulance or waiting-area data gathering	Licensed triage nurse retains acuity authority; staff verify any fields that could imply acuity
ED greeting and triage	Structured patient-entered data linked to evidence-based risk scores; patient goal and concern elicitation; dynamic queue and wait-time updates labeled as estimates	Narrative capture separated from acuity assignment; licensed triage nurse retains final triage decision
Posttriage and preclinician evaluation	LLM-enabled intake mapping spoken narrative to structured fields; personalized preventive screening and education micro-modules; voluntary virtual lower-acuity pathways	Role-stratified review (nurse or quality coordinator for low-risk; physician for diagnostic-adjacent outputs); EMTALA-compliant reversible pathways
Postevaluation communication	Condition-specific education reinforcement for new or incidental findings; closed-loop linkage to social-needs referrals	Clinician triggers and validates patient-facing content; social work or care management confirms referral receipt
Transition to hospitalization	Plain-language explanation of boarding steps; asynchronous patient Q&A while awaiting bed placement; expectation-setting for admission	Nursing sign-off on clinical content; clear escalation pathway for deterioration-related questions
Discharge	Literacy-leveled discharge instruction generation; medication counseling; red-flag symptom reinforcement	Clinician review and sign-off of all patient-facing text before release; no full automation of discharge communication
Postdischarge follow-up	Automated personalized check-ins; symptom escalation triage; medication-adherence and return-precaution reinforcement	Each outbound check-in mapped to a defined backend action owner (nurse coordinator, pharmacist, social worker)

ED, Emergency Department; EMTALA, Emergency Medical Treatment and Active Labor Act; LLM, large language model; PF-AI, patient-facing artificial intelligence; Q&A, question and answer.

### 2.1 Previsit

Before patients reach the triage desk, PF-AI can involve them directly in information gathering. Deployment channels include patient portals, mobile apps, and tablets in ambulances or waiting areas, each allowing patients to enter or verify information in real time. Pre-triage tools can analyze these inputs to guide patients toward the most appropriate care setting, potentially reducing unnecessary ED volume.^48^ Patients can also review key elements of their prehospital story so that handoffs to clinicians are both clinically accurate and patient-validated.^49^

Natural language processing pipelines convert patient-generated narratives into structured data for electronic health record integration.^50^, ^51^, ^52^, ^53^ A secondary benefit is confidentiality: patients can document sensitive information at their own pace rather than recounting it in a public triage area. A critical constraint applies. PF-AI should not delay safety-critical nursing triage or emergency stabilization. The practical model is to collect optional supplemental information after minimum triage safeguards are completed, then feed only validated, role-appropriate summaries into clinician workflows.^54^

### 2.2 ED Greeting and Triage

Triage sets the stage for patient trust and departmental flow.^55^ For patients able to participate, PF-AI may enhance the objectivity of triage by leveraging patient-entered data.^49^ These tools can capture information often missed before arrival, addressing limits on prehospital teams who may lack interoperable systems, reliable connectivity, or time for detailed documentation.^56^^,^^57^ The information can prepopulate portions of the Emergency Severity Index assessment to reduce repetitive questioning and ease nursing workload.^58^

Beyond data capture, PF-AI can transform triage into a more communicative and goal-oriented experience. Before clinician evaluation, these tools can elicit why the patient believes they are there, what worries them most, what outcomes they hope to achieve, and what values should guide their care. Capturing these priorities early allows clinicians to tailor discussions and align care plans with patient expectations, so that triage itself sets the stage for downstream flow and patient trust.

For participating patients, structured patient-entered data may improve consistency of history-taking, though any recommendations should be treated as decision support rather than autonomous triage decisions.^55^^,^^58^, ^59^, ^60^ Operationally, this requires separating patient narrative capture from acuity assignment. Patients can report goals, concerns, and barriers early, while licensed staff retain final authority over triage level, immediate testing priorities, and escalation pathways.

Transparent updates—such as current queue status and next steps—can reduce uncertainty, but messaging must avoid false precision. Wait-time estimates should, therefore, be clearly labeled as dynamic projections, and any high-risk symptom change should trigger immediate nurse reassessment rather than automated reassurance.

### 2.3 Posttriage and Preclinician Evaluation

Large Language Model (LLM)-enabled intake can collect richer patient narratives than generic symptom checkers by converting spoken accounts into structured fields mapped to evidence-based risk scores for clinician review.^61^, ^62^, ^63^, ^64^ LLMs can adapt questioning in real time based on health literacy or language preference,^65^^,^^66^ and organized outputs can be presented to the clinician to complete formal risk assessment and decision making.^67^

Additionally, PF-AI can support preventive screenings and educational engagement, for example through personalized micro-modules on chronic disease management.^68^, ^69^, ^70^ Emergency clinicians are necessarily focused on acute complaints, stabilization, and disposition, leaving limited opportunity for consistent preventive counseling. Under clinical oversight, PF-AI can automate parts of this work and shift it to waiting periods, adding value without delaying evaluation and helping ensure that health education and screening occur equitably across patient populations.^71^

Reliability safeguards are mandatory in high-volume settings. Recommended controls include constrained prompts, evidence-linked decision trees, schema-bound outputs, and physician-approved escalation logic before data enter actionable pathways. Because emergency physicians cannot reasonably review all generated content, oversight should be role-stratified: frontline nurses or quality coordinators review low-risk outputs, physicians adjudicate high-risk or discordant recommendations, and medical directors oversee periodic quality audits and model updates.

For lower-acuity pathways, virtual alternatives should remain voluntary, reversible, and compliant with Emergency Medical Treatment and Labor Act requirements. The expected benefit is reduced left-without-being-seen rates. However, systems must track whether digital diversion shifts burden onto patients with fewer resources.

### 2.4 Postevaluation Communication

PF-AI can also support structured, patient-centered communication about new and incidental findings. When diagnosing a new condition, clinicians can trigger PF-AI–assisted modules that reinforce key counseling points, expected treatment course, and follow-up needs. For incidental findings, PF-AI can deliver consistent, calm, and guideline-concordant explanations that emphasize next steps without alarmism, potentially reducing reliance on inconsistent web searches while keeping clinicians in control of the message.^72^ These same mechanisms can be extended to consent-related education for high-stakes diagnostics or procedures, where digital modules and comprehension checks complement, but do not replace, direct physician conversation.^73^

PF-AI can also connect clinical findings to social needs identified during intake.^74^ For example, when diagnosing an illness worsened by food insecurity, the clinician can refer the patient to a social work consultation and provide interactive information about local food banks. Linking medical and social interventions within the same workflow keeps the care plan actionable and the clinician central to coordination.

### 2.5 Transition to Hospitalization

The decision to hospitalize, and the subsequent wait for a bed, is often confusing and stressful for patients. PF-AI can support this transition through plain-language explanations of next steps, asynchronous question-and-answer channels for patients to raise concerns before the final discussion, and educational materials previewing what to expect during a hospital stay.^75^^,^^76^ When transfer to another facility is considered, the system can present information about the receiving hospital's resources and transportation options so that patients can make informed decisions.^77^^,^^78^ The objective at this stage is informed understanding of care pathways, not replacement of clinician counseling.^79^^,^^80^

### 2.6 Discharge

Discharge is a high-risk transition in which operational pressure routinely compromises clear communication.^10^ Discharge instructions commonly use medical language above most patients' reading levels.^7^^,^^10^ Generative AI can rewrite standard instructions in plain language and generate patient-specific audiovisual supplements (for example, short narrated videos or translated handouts), and recent studies show substantial gains in readability and comprehension.^7^^,^^9^^,^^10^

However, important safety limitations persist. Earlier LLMs produced discharge materials in which up to 18% of outputs contained potentially harmful inaccuracies. Newer models have reduced these risks through improved factual grounding, retrieval-augmented generation, and tighter integration with structured electronic health record data.^7^^,^^8^^,^^81^ Under clinician oversight, such models can now support a broader set of discharge tasks: context-aware tailoring of educational content, follow-up reinforcement, medication-adherence prompts, and connections to community resources.^82^^,^^83^

Full automation at discharge remains neither feasible nor advisable because discharge communication depends on clinician judgment, clinical reasoning, and interpretation of social context. The safest model is human-in-the-loop: generative AI drafts patient instructions, and clinicians review, edit, and contextualize content before release.^21^^,^^46^^,^^84^

### 2.7 Postdischarge Follow-Up

The period after discharge is one of the most vulnerable transitions in care, as patients move from a structured hospital environment to self-management at home.^85^ Breakdowns in coordination during this phase often lead to medication errors, missed follow-ups, and preventable readmissions.^86^ Common barriers include difficulties filling prescriptions, uncertainty about care instructions, and challenges with scheduling appointments or accessing ambulatory services due to limited transportation.^87^, ^88^, ^89^, ^90^

PF-AI may bridge these gaps through personalized, automated check-ins. AI can augment existing text message-based or telephonic follow-up interventions, which decrease ED revisits and hospital readmissions^6^^,^^91^ but can be slow, labor-intensive, and difficult to scale. PF-AI can also collect brief updates on symptoms, recovery progress, and daily function, giving clinicians a clearer view of how patients are doing after they leave the ED.^92^^,^^93^ Educational prompts can reinforce self-management, and reports on pain, fatigue, or wound healing can be summarized for care teams to identify patients who would benefit from further intervention. By maintaining open communication, PF-AI converts passive postdischarge follow-up into an ongoing partnership to support patient adherence, safety, and confidence during recovery.

Postdischarge automation is useful only when paired with a staffed response system. Every outbound check-in should map to a defined backend action owner (for example, nurse coordinator, pharmacist, social worker, or covering clinician) and response-time targets, so identified barriers trigger real intervention rather than unresolved alerts.

### 2.8 Operational Governance and Information Flow

Responsible PF-AI governance rests on three interdependent requirements: preserving information integrity, assigning accountability to specific roles, and verifying performance on an ongoing basis. Information flow across every touchpoint should follow a closed-loop design in which patient input is converted into a structured summary, risk-stratified, routed for role-based review, confirmed by a licensed clinician, and documented for downstream follow-through. Governance policies should specify who may release messages to patients, who is authorized to escalate safety concerns, what must be clinician-reviewed before any patient-facing output is released, and how every override is logged for subsequent quality review.

PF-AI is most accurately framed as a high-risk sociotechnical intervention rather than a discrete application deployment. Successful implementation, therefore, depends on transparent governance, explicit workflow ownership, and sustained institutional investment in safety monitoring. A recent national American College of Emergency Physicians membership survey documented meaningful institutional AI adoption among emergency medicine physicians (52%) alongside substantial uncertainty about bias (38%) and Health Insurance Portability and Accountability Act compliance (16%), underscoring the need for professional-society-backed governance norms.^94^ Strategic questions for emergency clinicians to pose to health-system leaders when evaluating, governing, and sustaining PF-AI deployments are provided in Table 2.

**Table 2:** Strategic questions for emergency physicians to ask health-system leaders.

Category	Strategic question
Protecting the bedside	How is this PF-AI deployment being sequenced relative to bedside investments (nurse staffing, interpreter services, social work)? What guarantees ensure that PF-AI spend does not displace direct patient-care capacity?
Physician wellness	What physician-wellness metrics (documentation burden, inbox volume, after-hours chart time, burnout inventory) will be tracked pre- and postdeployment, and who is accountable for reversing course if those metrics worsen?
Opt-out pathway	What is the opt-out pathway for patients (and for clinicians) who decline PF-AI mediation, and how is that pathway operationalized without penalizing access to care or clinician time?
Continuous oversight	Which multidisciplinary governance body owns ongoing oversight (clinical quality, equity, privacy, patient experience), how often does it review performance data, and what authority does it have to pause or terminate deployment?
Kill switch	What is the documented kill-switch procedure: who has authority to immediately disable PF-AI organization-wide, under what triggers, and what is the fallback workflow while the system is paused?

PF-AI, patient-facing artificial intelligence.

### 2.9 Challenges and Limitations

Workflow integration remains the single largest operational barrier. Fragmented tools generate duplicate work and contradictory documentation, whereas interoperable systems demand substantial build costs, ongoing maintenance, and standards alignment across vendors and health information exchanges. Strategic due-diligence questions for emergency clinicians to pose to PF-AI vendors are summarized in Table 3.^95^^,^^96^

**Table 3:** Strategic questions for emergency physicians to ask AI vendors.

Category	Strategic question
Confabulation guardrails	What are the specific technical guardrails against confabulation (formerly “hallucination”) in patient-facing outputs? Please describe the retrieval-augmented generation architecture, schema-bound output constraints, evidence-linked decision trees, and human-review requirements that prevent fabricated clinical content from reaching patients.
Xenomorphic design	How does the patient-facing interface communicate that patients are interacting with an automated system rather than a clinician? What nonanthropomorphic design cues, disclosure language, and identity disambiguation mechanisms are embedded, and how were these tested with patients from low digital-literacy backgrounds?
Medical incapacity	Under what clinical conditions is the system designed to explicitly disengage and escalate? Please list the medical-incapacity boundaries (eg, unstable vitals, altered mental status, active psychiatric crisis, pediatric high-acuity, pregnancy, and anaphylaxis) at which patient-facing outputs are suppressed and a licensed clinician is notified.
Click fatigue test	What is the mean number of additional clicks, fields, or screens an ED clinician must interact with per patient encounter when your PF-AI module is active, compared with baseline workflow? Please share any time-motion data from pilot sites.
Human-in-the-loop mechanics	Which roles (nurse coordinator, pharmacist, social worker, physician, medical director) receive which outputs at which decision points? What are the defined response-time targets for escalated outputs, and what happens when no human responds within the target window?
Algorithmic audits	What subgroup performance data—by race/ethnicity, language, age, disability status, and insurance—are generated and externally audited on a recurring cadence? Can institutions receive site-specific audit reports, and under what confidentiality terms?
Medico-legal accountability	Where does liability reside when a patient is harmed following a PF-AI output? Please describe the contractual allocation of liability among vendor, health system, and clinician, your indemnification terms, audit-log preservation policy, and cooperation commitments during litigation or regulatory inquiry.

ED, Emergency Department; PF-AI, patient-facing artificial intelligence.

Data protection obligations extend well beyond transport-layer security to full lifecycle governance: explicit consent language, granular access controls, defined retention limits, and user-facing controls that allow patients to disconnect data sources. Publicly available generative AI tools such as ChatGPT, Gemini, Llama, and Grok introduce additional Health Insurance Portability and Accountability Act exposure when used outside an institutional Business Associate Agreement; ED clinicians should verify platform coverage and consult privacy officers before entering any protected health information.^97^ Institutional trust also depends on rapid, transparent incident response when privacy events occur.

Liability remains unresolved when automated recommendations contribute to clinical action.^26^ Institutions should predefine accountability matrices spanning vendors, health systems, supervising clinicians, and operational review teams, with auditable logs for every high-risk recommendation and override decision. For high-risk recommendations—those affecting triage acuity, differential diagnosis in potentially unstable patients, or discharge of patients at risk for clinical deterioration—physician adjudication should be the designated endpoint of accountability.

A practical safety boundary is that PF-AI should not independently determine emergency triage level, final diagnosis, definitive treatment plans, or discharge of potentially unstable patients. Its role is to structure information, support communication, and surface concerns for licensed clinician decision making. This boundary is especially important given documented pre-existing disparities in human-assigned ED triage and disposition decisions, which any AI tool would otherwise inherit or amplify.^98^^,^^99^ Physician adjudication should be required for high-risk recommendations and safety overrides.

As part of a learning health system, programs should monitor process and outcome metrics: engagement, recommendation accuracy, override rates, clinician time burden, left-without-being-seen, return visits, follow-up completion, patient complaints, adverse events, and subgroup performance by language, literacy, race/ethnicity, insurance status, age, and digital access.^28^, ^29^, ^30^^,^^100^

Table 4 organizes these challenges as paired barrier-risk-safeguard triads spanning workflow integration, data protection, liability, safety boundaries, and performance monitoring. Equitable deployment, however, requires more than internal safeguards—it requires sustained codesign with the communities PF-AI will most affect. Drawing on community-based participatory research methodology, we frame this as a three-phase commitment. First, authentic partnerships are established before design begins: institutions identify patient communities most affected by the tool, engage community-based organizations as compensated partners rather than reactive reviewers, and surface the power asymmetries inherent in health-system-led development. Second, participatory codesign workgroups empower patient and community partners to shape tool behavior, interface design, and escalation logic across prepilot, pilot, go-live, and postdeployment phases, with explicit mechanisms to document and escalate dissenting perspectives when they conflict with clinical or operational priorities. Third, shared accountability metrics track equity-relevant outcomes—access, concordance, disparities in accuracy—on dashboards visible to community partners, who retain formal authority to trigger mitigation, pause, or sunset of a deployed tool and who receive plain-language reports on monitoring results. Table 5 provides strategic questions to operationalize each phase.

**Table 4:** Key barriers to PF-AI deployment and corresponding safeguards.

Barrier	Risk	Safeguard
Workflow integration	Fragmented tools create duplicate documentation, contradictory records, and added clinician time burden	Interoperable EHR-integrated deployment, pre-launch workflow mapping, and ongoing documentation-burden monitoring
Data protection and consent	Lifecycle data reuse may exceed patient expectations; insufficient access or retention controls	Explicit consent language, defined access controls, retention limits, and user-facing disconnect or delete options
Liability and accountability	Ambiguity when automated recommendations influence clinical action; responsibility diffuses across vendors, clinicians, and health systems	Predefined accountability matrix delineating responsibility across vendors, health systems, supervising clinicians, and end users
Safety boundary	Autonomous determination of triage acuity, diagnosis, treatment plan, or discharge of potentially unstable patients	PF-AI must not independently determine emergency triage level, final diagnosis, definitive treatment, or discharge of potentially unstable patients; licensed clinician retains final authority
Performance monitoring and equity	Model drift, subgroup performance gaps, unreported failures	Learning health-system surveillance of engagement, recommendation accuracy, override rates, clinician time burden, LWBS, and return visits, disaggregated by demographic subgroup

EHR, electronic health record; LWBS, left without being seen; PF-AI, patient-facing artificial intelligence.

**Table 5:** Strategic questions for co-designing PF-AI with patients and community-based organizations.

Category	Strategic question
Phase 1 — authentic partnerships	Which patient communities most affected by this PF-AI tool have been identified, and how were they identified? Which community-based organizations have been engaged as partners before design began, and what is the compensation structure for their time? How will power asymmetries between the health system and patient partners be surfaced and mitigated throughout the engagement?
Phase 2 — participatory codesign workgroups	What mechanisms allow patient and community partners to shape tool behavior, user interface, and escalation logic — not just provide reactive feedback? How are dissenting patient perspectives documented and escalated when they conflict with clinical or operational priorities? What is the cadence and structure of codesign sessions across the deployment lifecycle (prepilot, pilot, go-live, postdeployment)?
Phase 3 — shared accountability metrics	Which equity-relevant outcomes (access, concordance, disparities in accuracy) are monitored, and who sees the dashboards? What is the formal process for patient/community partners to trigger mitigation, pause, or sunset of a PF-AI tool? How are results of shared accountability monitoring reported back to participating communities in plain language?

PF-AI, patient-facing artificial intelligence.

## 3 Conclusion

The ED is an important but operationally demanding setting for responsible PF-AI. The strongest near-term opportunities lie in structured data transformation, multimodal communication support, and workflow augmentation—each implemented under explicit human oversight.

Adoption should proceed cautiously, with continued emphasis on reliability verification, equity protection, interoperability, cost sustainability, and legal accountability. Under this framework, PF-AI functions as an instrument for safer communication and coordination, although clinicians and care teams remain accountable for clinical decisions.

## Funding and Support

By *JACEP Open* policy, all authors are required to disclose any and all commercial, financial, and other relationships in any way related to the subject of this article as per ICMJE conflict of interest guidelines (see www.icmje.org). The authors have stated that no such relationships exist.

## Declaration of Generative AI and Ai-Assisted Technologies in the Manuscript Preparation Process

During the preparation of this work the authors used Anthropic Claude (Sonnet 4.5) and Google Gemini large language model tools, together with Model Context Protocol connectors to PubMed and ClinicalTrials.gov, in order to retrieve 2025 to 2026 peer-reviewed literature aligned to reviewer comments, draft structured candidate insertion text for iterative first-author review, assist in preparing tracked-change revisions, and help structure the response-to-reviewers document. After using these tools/services, the authors reviewed and edited the content as needed and take full responsibility for the content of the published article.
